# Effects of Dust Storms and Climatological Factors on Mortality and Morbidity of Cardiovascular Diseases Admitted to ED

**DOI:** 10.1155/2018/3758506

**Published:** 2018-07-02

**Authors:** Behcet Al, Mustafa Bogan, Suat Zengin, Mustafa Sabak, Seval Kul, M. Murat Oktay, Hasan Bayram, Ertan Vuruskan

**Affiliations:** ^1^Emergency Department, Medicine Faculty, Gaziantep University, Turkey; ^2^Biostatistics Department, Medicine Faculty, Gaziantep University, Turkey; ^3^Emergency Department, Medicine Faculty, Hasan Kalyoncu University, Turkey; ^4^Chest Disease Department, Medicine Faculty, University of Gaziantep, Gaziantep, Turkey; ^5^Cardiology Department, Medicine Faculty, University of Gaziantep, Turkey

## Abstract

**Objective:**

This study was designed to investigate the effects of Desert Dust Storms and Climatological Factors on Mortality and Morbidity of Cardiovascular Diseases admitted to emergency department in Gaziantep.

**Method:**

Hospital records, obtained between September 01, 2009 and January 31, 2014, from four state hospitals in Gaziantep, Turkey, were compared to meteorological and climatological data. Statistical analysis was performed by Statistical Package for the Social Science (SPSS) for windows version 24.0.

**Results:**

168,467 patients were included in this study. 83% of the patients had chest pain and 17% of patients had cardiac failure (CF). An increase in inpatient hospitalization due to CF was observed and corresponded to the duration of dust storms measured by number of days. However, there was no significant increase in emergency department (ED) presentations. There was no significant association of cardiac related mortality and coinciding presence of a dust storm or higher recorded temperature. The association of increases in temperature levels and the presence of dust storms with “acute coronary syndrome- (ACS-) related emergency service presentations, inpatient hospitalization, and mortality” were statistically significant. The relationship between the increase in PM10 levels due to causes unrelated to dust storms and the outpatient application, admission, and mortality due to heart failure was not significant. The increase in particle matter 10 (PM) levels due to causes outside the dust storm caused a significant increase in outpatient application, hospitalization, and mortality originated from ACS.

**Conclusion:**

Increased number of dust storms resulted in a higher prevalence of mortality due to ACS while mortality due to heart failure remained unchanged. Admission, hospitalization, and mortality due to chest pain both dependent and independent of ACS were increased by the presence of dust storms, PM10 elevation, and maximum temperature.

## 1. Background

Cardiac originated chest pain that is related or unrelated to the Acute Coronary Syndrome and heart failure are the most common cardiac diseases. ACS is a constellation of several complaints and signs caused by a disturbed equilibrium between myocardial oxygen supply and demand [1–4].

Heart failure has an unfavorable prognosis with one-year mortality rates of 5-10% and 30-40% for mild-to-moderate heart failure and severe heart failure, respectively. Six-year mortality rates following diagnosis are 80% for men and 65% for women. The survival rate after the development of pulmonary edema is potentially as low as 50%. The estimated one-week mortality risk following cardiogenic shock is greater than 85%. Acute cardiac failure may be comorbid with severe diseases such as arterial hypertension (53-73%), ischemic heart disease (46-68%), diabetes mellitus (DM) (27-42%), and atrial fibrillation (AF) (21-42%) [2–5].

Today, air pollution is well-known to have detrimental effects on human health. Inhalation of pollutants emitted in the atmosphere initiates a series of adverse effects that are detrimental to the overall well-being of residing inhabitants, particularly the respiratory and cardiovascular [6]. Fuels, which are utilized to maintain traffic activities, transportation, and to provide industrial and urban warming, are recognized as some of the primary sources of air pollution. Meteorological factors such as wind, temperature, pressure, and humidity can affect the distance of transportation and concentration of pollutants. The effects of pollutants on the environment and human health vary in relation to their chemical composition concentration and duration of sustained presence in the atmosphere. Significant adverse effects by air pollution on health especially respiratory and cardiovascular health have been overwhelmingly supported by numerous studies [3–8].

Epidemiological studies demonstrate that mortality and morbidity rates of cardiovascular diseases correlated with increased quantities of certain particular matter. However, the roles of desert dust storms and changes in climatological factors have not been assessed sufficiently. This study in Gaziantep, Turkey, was conducted to investigate the relationship between dust storms and increased levels of ≤ 10*μ*m particles (PM10) unassociated with dust storms and daily temperature levels (°C) with emergency service submissions, inpatient hospitalization, and mortality rates.

## 2. Methods

Ethics Committee approval of Gaziantep University Faculty of Medicine for this retrospective study was obtained on 23/06/2014 (decision no: 23.06.2014/228). The study was conducted in compliance with the Declaration of Helsinki protocols. A retrospective screening of the records dated between September 1, 2009, and January 31, 2014, was performed for patients, who presented to the emergency departments of Gaziantep University Şahinbey Research and Practice Hospital and three different state hospitals in Gaziantep (Dr. Ersin Arslan State Hospital, Şehit Kamil State Hospital, and 25th December State Hospital).

### 2.1. Creation of Groups and Inclusion Criteria

The obtained data was examined included the total population and four different subgroups. The subgroups were based on gender and age groups above and below 65 years. Patients older than 16 years of age who presented with heart failure and chest pain or diagnosed with acute coronary syndrome were included in the study.

### 2.2. Climatic Parameters and Particle Detection

Meteorological data was obtained as daily means, the maximum and minimum recorded temperature**s**, relative humidity, and air pressure (mbar). Daily levels of particulate matter of ≤ 10*μ*m were recorded as PM10, which refers, for this study, to particulate matter not originating from desert dust. Environmental data such as those of traffic, solid fuel use for warming, and industrial activities were obtained from local monitoring stations of Gaziantep City Centre for dates between January 31, 2014, and September 1, 2017. Data of desert dust storms in the district of Gaziantep were captured by photographic images from satellites provided by the link https://earthobservatory.nasa.gov. By utilizing this link, Aerosol Optical Depth (AOD) data was obtained for the period between the dates given above. Days when AOD was measured greater than 0.5 were categorized as stormy days based on support and advice by meteorologists.

### 2.3. Parameters for Comparisons

The relationship of the following factors, namely, admissions, hospitalization, and mortality was compared with the parameters defined as climate changes, dust storms, and particular substance levels.

### 2.4. Statistical Method

Normality of data was assessed by the Shapiro-Wilk test. According to the numerical variables, Mann–Whitney* U* test for non-normal data was used to compare dusty and nondusty days. Generalized additive regression models were built to investigate main and lag effects of PM10 (lag0 = first day of high level of PM10; lag1 = second day of high level of PM10; lag2 = third day of high level of PM10; and lag3 = fourth day of high level of PM10) and to assess other variables of cardiovascular mortality, including emergency department visits and hospitalization by adjusting possible confounding factors. Odds ratio (Ors) and confidence interval 95% (CI) estimates were used to show the direction of the effects. Relationship between meteorological parameters was investigated by using Spearman's rank correlation coefficient. Air pressure, relative humidity, and mean, minimum, and maximum temperatures were correlated to each other. To avoid multicollinearity problems in modeling, out of five variables, only maximum temperature, which was determined as the most statistically significant parameter in univariate analysis, was used in the models. Statistical analysis was performed by SPSS for windows version 24.0; statistical significance was determined by p<0.05.

## 3. Results

Throughout 1916 days, 86 dust storms were detected between September 2009 and February 2014. A total of 10 million hospital admissions to the emergency department were screened and 168,467 (1.68%) patients were included in the study.

**Figure 1:**
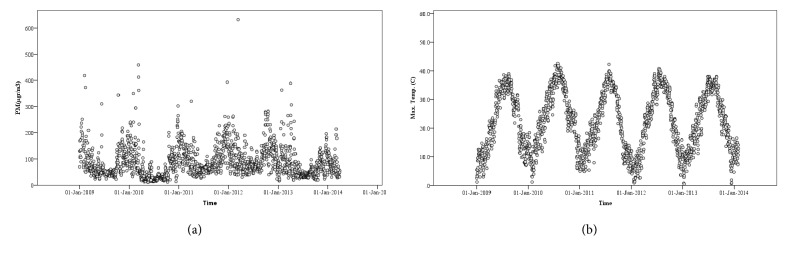
Daily mean of (a) particulate matter ≤ 10*μ*m (PM10) levels and (b) mean temperature (°C) in Gaziantep City Center, 01. 09.2009 to 31. 01. 2014.

Descriptive statistics and comparison of days with or without a dust storm for pollutants and meteorological variables during the study period are presented in Table 1. Mean, low, and high temperature values were significantly higher during dust storms (p < 0.001); however, air pressure values (mbar) were significantly lower when dust storms were present. No significant differences were found between PM levels and relative humidity. The trend of PM10 levels (Figure 1(a)) and the recorded high temperature values (Figure 1(b)) of Gaziantep City, 09.2009 to 31. 01.2014, was emphasized in Figure 1.

Of all patients, 29,047 patients (17%) had a diagnosis of CF, and 25,872 (89%) of them (12.792 females, 13.080 males) were discharged after ambulatory treatment. 12,627 (%49) all patients receiving ambulatory treatment were younger than 65 years of age. A total of 2042 (7%) patients (923 females, 1119 males) were admitted to inpatient services. From this group of patients, 1237 (60.58%) of them were over the age of 65. Mortality rate due to heart failure was 4% (n = 1133; 543 females, 590 males). 65.76% (n=745) of patients, who died of cardiac failure, were over the age of 65.

Inspection of the ambulatory presentation rates demonstrated lower values with PM10 lag0 and PM10 lag2 (p = 0.001/OR = 0.999). In females, hospital presentation rates were observed to be lower and these rates were associated with increased recorded high temperature values (p=0.010/ OR=0.997). The rates of inpatient hospitalization due to cardiac failure demonstrated lower figures with PM10 lag3 (p = 0.031) and with increased values of recorded high temperatures (p = 0.001). An increased rate of hospital presentations was observed on stormy days (p=0.001).

In our study, the parameters specified for the general population (PM10 levels, daily high temperature values, and days of the desert dust storm) demonstrated no effect statistically, on mortality due to cardiac failure (p > 0.05). However, investigation of subgroups revealed increased mortality rates for males with PM10 lag0 (p= 0.018) and PM10 lag3 (p = 0.028) (Table 2).

A total of 139,420 patients (83%) presented with chest pain. 29,703 (21.3%) of them were hospitalized upon ACS diagnosis, and the remaining patients underwent ambulatory treatment and were subsequently discharged. 2485 (8.3%) of all patients diagnosed with ACS (1091 female, 1394 male) had a fatal prognosis. In general, the mortality rate of chest pain in study patients, who presented with this complaint, was determined to be 1.8%. Twenty-nine percent of the patients (n = 8701) receiving a diagnosis of ACS were over 65 years of age.

Elevations of the recorded high temperature levels and presence of dust storm led to increased rates of patient presentations with chest pain (both in the general study population and in all subgroups) (p = 0.001). In the general population PM10 lag2 (p = 0.017) and PM10 lag3 (p = 0.016) led to an increase in outpatient presentations. In patients with ACS, a significant association between inpatient hospitalization and the following variables, namely, PM10 lag1 and PM10lag3 (p = 0.001), and the rise of temperature and dust storm (p= 0.001) was demonstrated.

The presence of dust storm (p = 0.001/OR = 0.400), PM10 lag0 (p = 0.037), PM10 lag2 (p = 0.003), and the recorded high temperature (p= 0.043) increased the mortality rates, as demonstrated statistically, in patients with ACS (Table 3).

## 4. Discussion

The presence of a dust mass in the air correlates with the days of a storm. According to various studies, the mass of dust measured during storm days was three to ten times greater than without storm days [9]. In recent years, a trend has been observed in the number of studies investigating the effects of dust storms on human health [10]. One of the correlations is the relationship of dust storms to chest pain and ACS. Studies done in the far East [4], Europe [5], and China [7], especially, reported significant findings. Throughout the world, studies on these issues have been pursued in non-emergency service facilities. However, the studies completed in Turkey and the Middle East are very limited in number despite the abundance of dust storms in these geographical locations. Our study is notable for both having been conducted at an emergency department and also being a more comprehensive study in that it investigates the effects of dust storms on emergency department visits due to chest pain and ACS, inpatient hospitalization, and mortality.

In general, the pathophysiology of ACS consists of mechanisms such as clotting/thrombosis, arrhythmias, acute arterial vasoconstriction, and systemic inflammatory responses [11]. Air pollution and dust clouds also act on those mechanisms leading to chest pain and ACS [12]. An experimental study on humans reported that ozone and fine particulate matter caused acute arterial vasoconstriction, leading to the development of ACS [13]. Another study reported that any increase of PM10 levels by 10 mcg/mm3 in individuals older than 65 caused elevated rates of hospital visits due to acute chest pain originating from cardiovascular factors [9]. The effects of PM10 levels can be sustained even though their levels are decreased to normal values. A study, confirming this fact, reported that on the 3rd day following the observed high levels of PM10, there was an increased number of hospital visits due to chest pain [14]. Our study reaffirmed the findings of these studies as well, by demonstrating a significantly increased number of hospital visits due to chest pain and ACS on PM10 lag2 and PM10 lag3 by the effect of PM10. Our study, in addition, reports decreased numbers of emergency service visits due to cardiac failure, a cardiovascular disease, on PM10 lag0 and PM10 lag2 despite the increase in the number of visits due to chest pain compared to those on PM10.

There are several studies investigating the effects of changes in air temperature levels on cardiovascular diseases which concluded nonspecific results. The most comprehensive one of those studies was pursued throughout twelve European cities [15]. This study reported a negative but nonsignificant association between temperature levels and hospital visits due to cardiovascular reasons. In contrast to this study, we observed that the presence of maximum temperature levels and storms caused a general increase in the number of emergency department visits due to chest pain.

Throughout the world, several studies have investigated the association between increased quantities of particulate matter and the rates of inpatient hospitalization. Nascimento et al. demonstrated an increased risk for cardiovascular diseases by an increase of 10 mcg/mm3 of particulate matter [14]. Ebrahimi et al. reported that, during the dust storms in Asia, an increase of PM10 by 100 mcg/mm3 caused increases in cardiovascular diseases, and the inpatient hospitalization was significantly increased on the 3rd day after exposure to the particulate matter (PM10 lag3) [16]. Onuzuka et al., too, reported decreased rates of inpatient hospitalization due to cardiovascular reasons at lower temperatures [17]. The results of our study demonstrated that the increase in temperature and the presence of dust storms led to significant increases in inpatient hospitalization due to ACS on PM10 lag1 and PM10 lag3. In addition, it was demonstrated that the presence of dust storms increased the hospitalization due to cardiac failure, whereas the increases in PM10 levels did not have an effect on this parameter.

There are many studies, which investigated the mortality associated with cardiovascular and respiratory problems during dust storms. All of these studies reported that dust storms increased mortality rates [18–20]. Kwon and Chen conducted a study reporting that Asian dust storms increased mortality rates due to cardiovascular and respiratory causes, although this finding was not statistically significant [19, 20]. Our study demonstrated increased rates of emergency department visits and hospitalization due to chest pain on dusty days but with statistically decreased rates of mortality. In addition, our study results did not show a significant association between the presence of dust storms and cardiovascular mortality.

### 4.1. Limitation

The leading limitations of this study are lack of data on the follow-up of patients following their emergency department visits after dust storms and no lag effect data on the third day after patient visits following the increased levels of PM10 not originating due to a storm. The retrospective nature of our study may also be a limiting factor.

### 4.2. Conclusion

Although there was an increase in admissions to the hospital due to heart failure on dusty days mortality was not affected. During periods when the maximum daily temperature increased, hospitalization due to heart failure decreased. Dust storms did not increase mortality due to heart failure; however, mortality due to ACS increased significantly. Admission due to chest pain that is associated/unassociated with ACS, hospitalization, and mortality was increased by the presence of dust storms, PM10 elevation, and maximum temperature. A person who is a whole in his environment must be more just and economical towards nature for the health of all living beings, both their own and their partners. The results from this study suggest that patients at risk should take extra precautions during dust storms or when air pollutants are present in high concentrations.

## Figures and Tables

**Table 1:** Descriptive statistics for pollutants and meteorological variables.

**Variables** **Median (IQR)**	**Overall** **(n = 1916 days)**	**Dust Storms Present** **(n = 89 days)**	**No Dust Storms** **(n = 1827 days)**	**p**
PM (*μ*g/m^3^)	72 [48-115]	74 [53.25-135]	72[48-114]	0.227
Mean temp (°C)	14.5[7.6-24.5]	21.8 [16.7-27]	13.9[7.3-24.2]	0.001*∗*
Max. Temp (°C)	21.8[12.6-31.8]	28.55 [23.42-34.57]	21.2[12.4-31.6]	0.001*∗*
Min. Temp (°C)	9.1[4-17.8]	16.1[10.8-21.3]	8.7 [3.9-17.6]	0.001*∗*
Relative humidity.	60.3[41-79]	53 [40.7-77.3]	60.85 [41-79.3]	0.288
Air pressure (mbar)	915.7 [912.3-919.75]	913.4 [910.8-916.05]	915.9 [912.4-919.9]	0.001*∗*

IQR: interquartile range; *∗* significant at the 0.05 level; Mann–Whitney *U* test

**Table 2:** Results of generalized additive Poisson models for predicting number of emergency department visits and hospitalization due to CCF.

**Variables**		**Admission** **OR [95% CI] **^**a**^	**p**	**Hospitalization** **OR [95% CI] **^**a**^	**p**	**Mortality** **OR [95% CI] **^**a**^	**p**
TOTAL	PM0	0.999 [0.999-1.000]	0.001*∗*	0.999 [0.998-1.000]	0.148	1.001 [1.000-1.003]	0.127
	PM1	1.000 [1.000-1.001]	0.348	0.999 [0.997-1.000]	0.077	0.998 [0.996-1.000]	0.116
	PM2	0.999 [0.999-1.000]	0.025*∗*	1.000 [0.998-1.002]	0.988	1.000 [0.998-1.002]	0.720
	PM3	1.000 [0.999-1.000]	0.345	0.999 [0.997-1.000]	0.031*∗*	1.001 [1.000-1.003]	0.162
	MT	0.999 [0.997-1.000]	0.094	0.981 [0.976-0.987]	0.001*∗*	1.005 [0.998-1.012]	0.199
	DS	1.003 [0.972-1.036]	0.833	2.209 [2.069-2.359]	0.001*∗*	0.956 [0.819-1.115]	0.564

WOMEN	PM0	0.999 [0.998-0.999]	0.001*∗*	0.999 [0.997-1.001]	0.180	1.000 [0.997-1.002]	0.784
	PM1	1.000 [1.000-1.001]	0.607	0.999 [0.997-1.001]	0.487	0.999 [0.996-1.002]	0.639
	PM2	1.000 [0.999-1.000]	0.281	0.999 [0.997-1.002]	0,527	1.001 [0.999-1.004]	0.361
	PM3	1.000 [0.999-1.000]	0.795	0.999 [0.997-1.001]	0.366	1.000 [0.997-1.002]	0.856
	MT	0.997 [0.995-0.999]	0,010*∗*	0.982 [0.975-0.999]	0.001*∗*	1.008 [0.998-1.018]	0.120
	DS	0.960 [0.915-1.007]	0.091	2.109 [1.909-2.330]	0.001*∗*	0.907 [0.718-1.146]	0.413

MEN	PM0	0.999 [0.999-1.000]	0,034*∗*	0.999 [0.998-1.001]	0.463	1.002 [1.000-1.004]	0.018*∗*
	PM1	1.000 [1.000-1.001]	0.400	0.998 [0.996-1.000]	0.081	0.998 [0.995-1.001]	0.123
	PM2	0.999 [0.999-1.000]	0,036*∗*	1.001 [0.990-1.003]	0.543	0.998 [0.995-1.001]	0.189
	PM3	1.000 [0.999-1.000]	0.289	0.998[0.996-1.000]	0.037*∗*	1.002 [1.000-1.004]	0.028*∗*
	MT	1.000 [0.998-1.002]	0.840	0.981 [0.974-0.998]	0.001*∗*	1.001 [0.992-1.011]	0.780
	DS	1.043 [0.999-1.089]	0.055	2.292 [2.100-2.502]	0.001*∗*	0.992 [0.808-1.218]	0.938

> 65 years	PM0	0.999 [0.999-1.000]	0.029*∗*	0.999 [0.998-1.001]	0.363	1.001 [0.998-1.003]	0.378
	PM1	1.000 [0.999-1.000]	0.471	0.998 [0.996-1.000]	0.132	0.999 [0.996-1.002]	0.483
	PM2	0.999 [0.999-1.000]	0.070	1.000 [0.998-1.002]	0.895	0.999 [0.998-1.001]	0.302
	PM3	1.000 [1.000-1.001]	0.740	0.999 [0.998-1.001]	0.275	1.002 [1.000-1.004]	0.077
	MT	0.999 [0.997-1.001]	0.376	0.983 [0.976-0.989]	0.001*∗*	1.003 [0.995-1.012]	0.434
	DS	0.972 [0.929-1.018]	0.232	2.180 [2.004-2.371]	0.001*∗*	1.012 [0.845-1.212]	0.896

< 65 yr.	PM0	0.999 [0.998-0.999]	0.001*∗*	0.999 [0.997-1.001]	0.241	1.002 [0.999-1.005]	0.149
	PM1	1.001 [1.000-1.001]	0.038*∗*	0.999 [0.996-1.001]	0.346	0.997 [0.993-1.000]	0.088
	PM2	1.000 [0.999-1.000]	0.183	1.000 [0.998-1.003]	0.836	1.001 [0.998-1.005]	0.380
	PM3	1.000 [0.999-1.000]	0.092	0.998 [0.996-1.000]	0.035*∗*	1.000 [0.997-1.003]	0.966
	MT	0.998 [0.996-1.001]	0.138	0.979 [0.971-0.998]	0.001*∗*	1.007 [0.995-1.019]	0.265
	DS	1.035 [0.990-1.082]	0.130	0.907[2.030-2.506]	0.413	0.836 [0.622-1.124]	0.236

DS: dust storm adjusted odds ratios and 95% confidence intervals; *∗* significant at 0.05 levels; CCF: congestive cardiac failure

**Table 3:** Results of generalized additive Poisson models for predicting the number of emergency department visits and hospitalization due to ACS.

**Variables**		**Admission** **Or [95% CI] **^**a**^	**p**	**Hospitalization** **Or [95% CI] **^**a**^	**p**	**Mortality** **Or [95% CI] **^**a**^	**p**
TOTAL	PM0	1.000 [1.000-1.000]	0.498	1.000 [1.000-1.001]	0.098	1.001 [1.000-1.002]	0.037*∗*
	PM1	1.000 [1.000-1.000]	0.157	1.001 [1.000-1.001]	0.001*∗*	0.999 [0.998-1.001]	0.293
	PM2	1.000 [1.000-1.000]	0.017*∗*	1.000 [0.999-1.000]	0.132	1.002 [1.001-1.003]	0.003
	PM3	1.000 [1.000-1.000]	0.016*∗*	1.002 [1.001-1.002]	0.001*∗*	1.000 [0.999-1.001]	0.919
	MT	1.004 [1.003-1.005]	0.001*∗*	1.005 [1.004-1.007]	0.001*∗*	1.005 [1.000-1.009]	0.043*∗*
	DS	1.150 [1,135-1.166]	0.001*∗*	1.304 [1.273-1.336]	0.001*∗*	0.400 [0.316-0.505]	0.001*∗*

WOMEN	PM0	1.000 [1.000-1.000]	0.844	1.000 [1.000-1.001]	0.447	1.000 [0.998-1.002]	0.932
	PM1	1.000 [1.000-1.001]	0.023*∗*	1.001 [1.000-1.001]	0.008*∗*	1.000 [0.998-1.002]	0.986
	PM2	1.000 [1.000-1.000]	0.027*∗*	1.000 [0.999-1.000]	0.295	1.001 [0.999-1.003]	0.172
	PM3	1.000 [1.000-1.000]	0.101	1.002 [1.001-1.002]	0.001*∗*	1.001 [0.999-1.002]	0.379
	MT	1.004 [1.004-1.005]	0.001*∗*	1.003 [1.001-1.006]	0.012*∗*	1.004 [0.997-1.011]	0.221
	DS	1.129 [1.109-1.150]	0.001*∗*	1.286 [1.232-1.343]	0.001*∗*	0.410 [0.289-0.582]	0.001*∗*

MEN	PM0	1.000 [1.000-1.000]	0.221	1.000 [1.000-1.001]	0.336	1.002 [1.001-1.004]	0.004*∗*
	PM1	1.000 [1.000-1.000]	0.703	1.001 [1.000-1.001]	0.004*∗*	0.999 [0.997-1.001]	0.163
	PM2	1.000 [1.000-1.000]	0.267	1.000 [0.999-1.000]	0.090	1.002 [1.001-1.004]	0.006
	PM3	1.000 [1.000-1.000]	0.074	1.002 [1.001-1.002]	0.001*∗*	1.000 [0.998-1.001]	0.523
	MT	1.004 [1.003-1.005]	0.001*∗*	1.003 [1.001-1.005]	0.002*∗*	1.005 [0.999-1.011]	0.107
	DS	1.175 [1.152-1.198]	0.001*∗*	1.360 [1.317-1.404]	0.001*∗*	0.390 [0.285-0.534]	0.001*∗*

> 65 years	PM0	1.000 [0.999-1.000]	0.267	1.000 [1.000-1.001]	0.447	1.001 [1.000-1.003]	0.063
	PM1	1.000 [1.000-1.001]	0.601	1.001 [1.000-1.001]	0.008*∗*	0.999 [0.997-1.001]	0.245
	PM2	1.000 [0.999-1.000]	0.199	1.000 [0.999-1.000]	0.295	1.002 [1.000-1.003]	0.012*∗*
	PM3	1.001 [1.000-1.001]	0.002*∗*	1.002 [1.001-1.002]	0.001*∗*	1.000 [0.998-1.001]	0.618
	MT	1.004 [1.003-1.006]	0.001*∗*	1.003 [1.001-1.006]	0.012*∗*	1.000 [0.994-1.006]	0.917
	DS	1.281 [1.245-1.135]	0.001*∗*	1.286 [1.232-1.343]	0.001*∗*	0.376 [0.270-0.523]	0.001*∗*

< 65 yr.	PM0	1.000 [1.000-1.000]	0.187	1.000 [1.000-1.001]	0.133	1.001 [0.999-1.003]	0.283
	PM1	1.000 [1.000-1.000]	0.191	1.001 [1.000-1.001]	0.011*∗*	1.000 [0.998-1.001]	0.790
	PM2	1.000 [1.000-1.000]	0.044*∗*	1.000 [0.999-1.000]	0.269	1.002 [1.000-1.003]	0.100
	PM3	1.000 [1.000-1.000]	0.228	1.002 [1.001-1.002]	0.001*∗*	1.001 [0.999-1.001]	0.451
	MT	1.004 [1.003-1.005]	0.001*∗*	1.007 [1.005-1.006]	0.001*∗*	1.011 [1.004-1.006]	0.003*∗*
	DS	1.118 [1.101-1.135]	0.001*∗*	1.312 [1.275-1.343]	0.001	0.428 [0.308-0.523]	0.001*∗*

DS: dust storm; a: adjusted odd ratios and 95% confidence intervals; *∗*: significant values;*∗* significant at 0.05 level.

## Data Availability

The data used to support the findings of this study are available from the corresponding author upon request.

## Abbreviations

SPSS: Statistical Package for the Social Science
CF: Cardiac failure
PM: Particles matter
ED: Emergency department
ACS: Acute coronary syndrome
DM: Diabetes mellitus
AF: Atrial fibrillation
AOD: Aerosol Optical Depth
CI: Confidence interval
Ors: Odds ratio.
